# What’s up doc? Physicians’ reflections on their sustainable employability throughout careers: a narrative inquiry

**DOI:** 10.1186/s12913-024-10924-1

**Published:** 2024-04-26

**Authors:** Iris van de Voort, Irene Grossmann, Ian Leistikow, Jan-Willem Weenink

**Affiliations:** 1 Erasmus School of Health Policy and Management, Erasmus University Rotterdam, Oudlaan 50, Rotterdam, 3062 PA The Netherlands; 2 Center for Safety in Healthcare, Institute for Health Systems Science at TPM Faculty, Delft University of Technology, Delft, The Netherlands; 3 Dutch Health & Youth Care Inspectorate, Ministry of Health, Welfare & Sport, Utrecht, the Netherlands

**Keywords:** Physicians, Sustainable employability, Employment context, Self-regulation, Physician wellbeing, Healthcare quality

## Abstract

**Background:**

Physicians have complex and demanding jobs that may negatively affect their sustainable employability (SE) and quality of care. Despite its societal and occupational relevance, empirical research on physicians’ SE is scarce. To further advance our understanding of physicians’ SE, this study explores how physicians perceive their employment context to affect their SE, how physicians self-regulate with the intent to sustain their employability, and how self-regulations affect physicians’ SE and their employment context.

**Methods:**

Twenty Dutch physicians from different specialisms were narratively interviewed between March and September 2021 by a researcher with a similar background (surgeon) to allow participants to speak in their own jargon. The interviews were analyzed collaboratively by the research team in accordance with theory-led thematic analysis.

**Results:**

According to the interviewees, group dynamics, whether positive or negative, and (mis)matches between personal professional standards and group norms on professionalism, affect their SE in the long run. Interviewees self-regulate with the intent to sustain their employability by (I) influencing work; (II) influencing themselves; and (III) influencing others. Interviewees also reflect on long-term, unintended, and dysfunctional consequences of their self-regulations.

**Conclusions:**

We conclude that physicians’ SE develops from the interplay between the employment context in which they function *and* their self-regulations intended to sustain employability. As self-regulations may unintentionally contribute to dysfunctional work practices in the employment context, there is a potential for a vicious cycle. Insights from this study can be used to understand and appraise how physicians self-regulate to face complex challenges at work and to prevent both dysfunctional work practices that incite self-regulation *and* dysfunctional consequences resulting from self-regulations.

## Background

Physicians’ sustainable employability (SE), defined as their “ability to function [adequately] at work and in the labor market” throughout working lives [1], is threatened due to aging populations, increased retirement ages, and personnel shortages in health care [2, 3]. As a result, physicians experience heavier workloads, more time constraints, and increased work pressure [4]. This adds to job demands that are already physically, mentally, and emotionally demanding, such as bearing high responsibility; regularly working overtime; experiencing adversity; and being exposed to disruptive behavior from both patients and colleagues [5–8]. In addition, physicians who practice independently engage in many nonclinical tasks, such as education, research, management, and quality improvement work, which are not always fully compensated for time or salary [9]. Although physicians may be trained or sensitized to such job aspects, over time these may overshadow the fulfilling and valued parts of a career in medicine, in turn challenging physicians’ SE [10, 11].

The medical education community previously warned of a “wellness crisis in medicine” with alarmingly high prevalence rates of burn-out, depression, anxiety, impaired empathy, and job dissatisfaction [12]. Over time, poor well-being and impaired health are associated with a compromised ability to function adequately at work (i.e., SE) [1]. SE issues are not only disruptive to physicians personally and professionally, but also affect the quality and safety of health care through the provision of suboptimal care, decreased patient satisfaction, and medical errors [13–15]. In addition, perceiving one’s ability to function as suboptimal may incite a vicious cycle of stress, impairment, and again, greater odds of making a future mistake [16]. In sum, sustaining physicians’ employability is imperative for both physicians and patients, and, as some argue, should become a “non-negotiable” pre-condition for any health care system that aspires to deliver safe, patient-centered, efficient, and effective care [17].

As a concept, SE has been extensively theorized and debated, despite being an emerging body of literature (for conceptual reviews see: [1, 18]. Despite conceptual abundance in SE research, peer-reviewed international empirical research on physicians’ SE is scarce, which is surprising given its occupational and societal relevance in many settings. A notable exception includes the research of van Leeuwen (et al.), who examined the role of context (i.e., the COVID-19 pandemic) [19], job characteristics (i.e. job autonomy) [20], and job crafting [21] in relation to physicians’ SE. Although insightful, these are all quantitative studies, and as such, there appears to be a gap in the literature in terms of understanding physicians’ own experiences and perceptions derived from in-depth qualitative research. Qualitative data from physicians’ stories may enhance a contextualized understanding of physicians’ SE, pave the way for more explanatory theories, and inform appropriate policy and intervention [22].

In this interview study we sought to fill that gap by interviewing mid- and late-career physicians about their SE. The main question guiding our research is how physicians perceive their SE, and how they sustain their employability throughout careers. In the next section, we present our theoretical framework in which we gradually introduce the subquestions of this research as well as the concepts that guide our analysis.

## Theoretical background

### Sustainable employability

The term SE first originated in policies to stimulate more and prolonged work participation among the working population in the face of ageing populations. Ageing populations threaten the financial viability of social security and retirement policies and are likely to result in labour market shortages in societally valued sectors, e.g. healthcare. As a result, a public interest developed to stimulate the working population to continue work until, or even after, retirement [23]. Ageing populations particularly affect the health care sector because they translate to an increased demand for patient care that will have to be accommodated by fewer health care workers, as many will retire in the future [24]. These societal developments make the SE of healthcare workers, including physicians, a pressing societal issue.

In academic literature, SE is a frequently used concept, but it is conceptualized and operationalized in equally frequent ways, leaving the field of SE research quite “scattered” [25]. Theoretical literature on SE can be roughly categorized into three strands, each conceptualizing SE differently. First, the concept is often utilized to capture an individual’s labor market opportunities, chances, or potential [26]. Second, SE may refer to the extent to which employees are willing and able to continue work (often until retirement) [27]. This conceptualization is often used in the context of retaining employees in organizations or sectors, particularly in sectors that face increasing personnel shortages, such as the health care sector. Third, scholars use the concept in an extended manner by arguing that it is not only about having employment but also about how well an individual is able to function at work [1]. In other words, the extent to which employees are able to function “as effectively, efficiently, and healthily as possible within a given (un)employment context, now and in the future” [28]. According to this perspective, individuals who are employed may still not be sustainably employable if their ability to function adequately at work is negatively affected by that employment over time [1]. As such, SE also refers to the quality of employment. In relation to physicians’ SE, empirical work is mostly conducted on the basis of the second conceptualization. In the next section, we will argue why the third strand of SE conceptualizations may be better suited to understanding physicians’ SE.

Mid-, and late-career physicians are more likely to face difficulties to their SE in terms of functioning adequately at work throughout their careers (i.e., third strand) because of high job demands (e.g., hectic workloads) and limited job resources (e.g., lack of meaningful contact with patients) [29]. It is less likely that physicians will struggle to find employment (i.e., first strand) or will not retain employment in medicine (i.e., second strand). Practising physicians generally do not have difficulty finding or keeping jobs, not even in times of economic downturn [13]. Moreover, while some studies find alarmingly high rates of physicians’ *intent* to leave medicine, the number of physicians who actually leave medicine altogether, excluding those involved in normal retirement, is estimated to be low [30]. This is probably due to their highly specialized knowledge, which is not easily transferable to other jobs and their considerable investment to become a physician [3]. The few physicians who do leave medicine are probably offset by a growing trend of physicians who continue working beyond the traditional retirement age [31]. In addition, despite widespread ill-health (notably burn-out), sickness absence rates of physicians do not tend to be high, while presenteeism (i.e., working with health complaints) is considerably higher, again suggesting that physicians’ labor market participation is not the issue here [32].

In sum, we argue that researchers on SE should adopt a conceptualization that is most relevant to their population under study. Given the above, we posit that there should be more empirical research that is built on the third strand of SE conceptualizations. To date, existing empirical work on physicians’ SE [19–21] has, however, focused on the second strand. As such, this study continues based on the premises of the third strand of SE conceptualizations, specifically focusing on physicians’ long-term ability to function adequately at work.

### Employment context

Many SE scholars have underlined the importance of someone’s employment context for understanding their SE [1, 11, 33]. Fleuren et al. further stress the need to theoretically distinguish between SE as an individual-level construct on the one hand, and contextual factors as potential antecedents to SE on the other hand [34]. The authors propose that an individual’s employability can be called sustainable if it has not been negatively affected by that person’s employment over time, in which case the latter may be named ‘sustainable employment’ [1]. Relevant contextual factors may be present at the individual, work, team/group, organizational, professional, cultural and societal level, together constituting someone’s employment context [1]. The relevance of contextual factors is exemplified by a longitudinal survey that demonstrated that physicians perceived their own SE to improve during the COVID-19 pandemic compared to before, which could have been the result of “an increase in societal appreciation” [19]. Other important contextual SE factors - although not specific to physicians - include the importance of certain HR practices (e.g. development opportunities) [35] or organizational climate [36]. More qualitative research may uncover which specific contextual factors, as perceived by physicians, affect physicians’ SE throughout careers.

In addition, several theories may offer an explanation as to *how* contextual factors affect someone’s SE. First, Klink et al. argue that contextual factors are important because they explain to what extent workers can realize value from work [11]. Examples of values that can be attained from work in a given context include a sense of security, meaning, recognition, structure in life, personal identity, feeling needed in society, self-esteem, social contacts, or possibilities to learn [37]. For physicians, practicing medicine provides a space for intellectual stimulation, an opportunity to help others, and a way to be meaningful to society [38]. To advance our understanding of SE, Klink et al. recommend that researchers examine what employees value at work in a particular context and to what extent they are enabled and able to achieve these values [11]. Second, the person-environment fit perspective, as applied by Van Vuuren, van der Heijden, and Semeijn in their research on the SE of university staff members, proposes that employees are more likely to be sustainably employable if there is a fit between the employee (i.e., person) and their employment context (i.e., environment) [36]. The authors illustrate the person-environment fit with an example: an individual’s SE can be enhanced if employees’ natural inclination to develop is facilitated by an employment context that stimulates learning. To connect both perspectives, a P-E fit is likely to arise if employees are enabled by their work context to realize value from work, whilst employees are simultaneously willing to materialize these opportunities [36].

In sum, given the importance of the employment context to physicians’ SE, the first subquestion guiding our study is: “How do physicians perceive their employment context to affect their SE?”

### Self-regulation

Another limitation in research concerning individuals’ SE is that most conceptual models seem to presume a passive role for individuals towards their employment context and neglect their opportunity and ability to self-regulate if and how their SE is affected by the employment context [39]. Luckily, more research has become available that acknowledges the active agency of modern workers: “The average worker has developed from a more-or-less passive performer of predefined tasks to an increasingly autonomous and responsible entrepreneur in his or her work (“intrapreneur”), who proactively sets his/her own goals and makes his/her own choices and (shared) decisions).” [11] In the context of this research, the concept of self-regulation may be helpful for understanding physicians’ agency in sustaining their employability [28].

Self-regulation entails any conscious or unconscious attempt by the ‘self’ to regulate (i.e. plan, generate, control, and/or adjust) [40] “thoughts, feelings, and actions in order to achieve personal goals and adapt to one’s changing environment.” [41] Self-regulation requires three ingredients to ‘work’, namely, standards, monitoring, and operation [42]. First, self-regulation requires a commitment to standards, “for goal-directed behavior [e.g. self-regulation] is impossible without a goal”. Personal standards– synonymous with values, goals, ideals, and/or expectations– can be intrinsically or extrinsically shaped (i.e. by oneself or others). Second, self-regulation requires monitoring processes to determine progress toward achieving standards. Third, self-regulation requires the capacity to make changes (i.e. to operate). This capacity draws upon a limited resource that can become depleted if not conserved, replenished, or strengthened [42]. According to Baumeister, Schmeiche and Vohs, individuals aspire to achieve a better ‘fit’ between themselves and their environment (in line with P-E fit theory) through primary or secondary control strategies [42]. Primary strategies are targeted at changing the environment to align with the self, and secondary strategies are aimed at changing the self to conform to the environment. When converting these three ingredients to the context of SE, it is likely that self-regulation is employed by employees to achieve a certain standard (here: being sustainably employable), monitor the extent to which their ability to function deviates from that standard (here: actual versus optimal/adequate functioning), and accordingly take action to eliminate differences. In brief, self-regulation may be used to “strive for optimal functioning (…) [and] “to cope with adversity and dysfunction.” [41] 

The importance of self-regulation for individuals’ SE is widely acknowledged in SE literature [28, 43], but scholars have used different terms to capture this agentic, autonomous and goal-directed behavior of workers (e.g., ‘career self-directedness’ [44], ‘career self-management’ [45], and ‘self-leadership’ [46]. Scholars reason that employees who regulate effectively are more likely to sustain positive states (e.g., well-being or SE) in the event of adversity [41, 47]. Conversely, compromised SE may be partially explained by ‘regulatory failure’, either due to underregulation (i.e. not engaging in self-regulation) or misregulation (engaging in ineffective self-regulation) [48]. To date, only a few scholars have conducted studies on the self-regulation of physicians. These studies conclude that high self-regulating physicians also report higher well-being levels than low self-regulating physicians [41, 49]. The authors reason that physicians with high self-regulating capacity are likely to “maintain focus on what is important to them, especially in the face of obstacles, […] preserve a sense of purpose in their work”, and engage in self-acceptance [49]. To date, however, there is no available research that describes in detail how physicians exactly self-regulate with the intent of sustaining their employability, or how these self-regulations emerge and develop. It is likely that physicians’ self-regulations are “context-specific processes” [50] that are heavily influenced by culturally embedded norms in medicine that stipulate how physicians are supposed to behave or what they ought to strive for [51]. For example, taking sick leave, which could be considered self-regulation targeted at SE (i.e. recovering to sustain one’s ability to function adequately thereafter) is surprisingly low among physicians [52, 53]. This may be because taking sick leave clashes with “the ‘ideal worker’ role, in which physicians are available to work full time (…) without significant family obligations” [54]. In contrast, presenteeism - the choice to continue working while ill or impaired—is very prevalent, perhaps because this self-regulating behavior does align with norms in medicine [55].

In conclusion, the fields of SE and self-regulation may benefit from an in-depth account of physicians’ self-regulations. Accordingly, we present our second subquestion: “how do physicians self-regulate with the intent to sustain their employability?”

### Consequences of self-regulations

In addition, very little is known about the short- and long-term consequences of physicians’ self-regulations, other than that physicians with high self-regulating capacity report higher levels of psychological wellbeing than physicians with low self-regulating capacity [41]. It is likely that self-regulations, although well-intended and perhaps functional in the short-term, may still bring about “dysfunctional consequences” in the long-run or unintentionally feedback into the employment context [32]. For example, when sick leave is averted, there may be a risk for additional or chronic impairment, making errors at work, and maintaining presenteeism cultures [32]. More empirical research is needed to understand how self-regulations unfold in practice in the long run. Accordingly, we present our third subquestion: “How do physicians’ self-regulations affect their SE and their employment context?”.

**Fig. 1:**
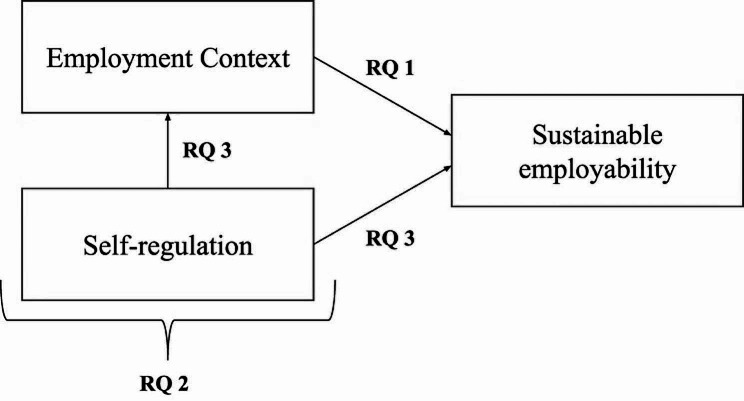
Conceptual model and research questions

To conclude, we have explained how physicians deal with demanding work practices in a complex, layered employment context that likely affects their SE over time. We have argued that it is likely that physicians try to sustain their employability through self-regulation but that the nature, origin, rationale, and consequences of these regulations remain largely unknown. In sum, this study seeks to answer the following research questions (Fig. 1: provides a schematic depiction of the research questions):

I: How do physicians perceive their employment context to affect their SE?

II: How do physicians self-regulate with the intent to sustain their employability?

III: How do physicians’ self-regulations affect their SE and their employment context?

## Methods

### Data collection

In this study we focused on physicians’ *subjective* understanding of their SE. We allow participants to explore, elaborate and construct their own stories on what it means to function adequately at work in a particular context. This method is in line with narrative inquiry which aims to “move away from traditional ways of knowing and telling in the social sciences towards multiple ways of knowing and telling, away from traditional quests for objectivity towards a celebrated acceptance of subjectivity, away from grand narratives towards local narratives and away from facts and towards meaning.” [56] Narrative inquiry zooms in on personal and unique experiences, but as it asserts that individuals operate within, and are part of, a wider social context, it also offers researchers the possibility to learn about the ‘universal’ [57]. To help participants construct their own stories, there was no predefined structure for the interviews, other than introducing the background of the research team and our research aims. As we expected some apprehensiveness in disclosing stories, we reasoned that an interviewer who (had) worked as a physician and was familiar with the culture and organization of physician work would enable interviewees to talk in their own jargon, as they would among peers. To establish trust and confidentiality, we decided to conduct the interviews as a dialogue, where the interviewer also disclosed personal stories on her experiences performing as a surgeon. Every interviewee was informed about the aim of the study before the interview by e-mail and in person. Consent to record, transcribe, analyse, and report their stories was obtained and recorded before the start of the interview. Physicians could indicate a preferred location for the interviews. The interviews lasted between 50 and 110 min and were audiotaped and transcribed anonymously with the help of student assistants.

### Interviewees

A total of twenty Dutch physicians were interviewed between March and September 2021. We specifically focused on physicians who finished training and residency, as we believe this to be a very distinct career phase with distinct challenges, such as carrying final responsibility over performance and engaging in nonclinical tasks. Initially, physicians were recruited via the network of the research team, either because these participants had a known affinity with the topic (e.g., in research or practice) or because they were known to have had a pleasant or unpleasant experience in their careers that had affected their SE. These interviewees introduced us to new potential interviewees. We continued recruiting physicians until we reached saturation. In total we interviewed 12 male and 8 female physicians, ranging from recently started physicians (< 5 years) to physicians who had been retired for some time. Nineteen physicians are medical specialists (e.g., surgeon or psychiatrist) and one works as a general practitioner.

### Setting

In the Netherlands, 70% of medical specialists are employed by health care organizations, and 30% are self-employed [58]. Self-employed physicians in hospitals are usually members of Medical Specialist Companies (MSC’s), an assemblage of “multiple mono-disciplinary specialty groups” that have varying degrees of interdisciplinary collaboration and are governed by a chosen group of peers [59]. GPs tend to work self-employed (72% in 2021) and either have their own practice or are hired as self-employed GPs by another practice for a fixed or temporary period of time [60].

### Data analysis

We conducted a theory-led form of thematic analysis [61] in which we used our conceptual framework and the research questions to organize codes, which were inductively generated, into the following story elements: (I) physicians explaining how their employment context impacted their SE; (II) physicians narrating how they had self-regulated their SE; and (III) physicians reflecting on the consequences of their self-regulations. The coding scheme was developed in Atlas.ti through collaborative data analysis because we reasoned that an insider (IG) and an outsider (IV) perspective would limit biases from both standpoints. We followed the six steps formulated by Richards and Hemphill [62], who offer a structured and transparent approach to analysing qualitative data with multiple researchers, with explicit steps to engage in dialogue and discussion to reach consensus throughout the process. IV and IG both coded the interviews. Initial and evolving codes and schemes were presented to IL and JW, and as a team we discussed and fine-tuned the codes, categories, and themes for subsequent analysis. In determining what themes to report, we decided to utilize our insider/outsider perspective again. From the insider perspective, the included themes had to be recognizable, deemed imperative, and ideally represent something that has currently received little attention in practice. We also e-mailed our participants with preliminary results to ensure that these requirements were met. From the outsider (here: theoretical) perspective, we considered themes to be worth reporting upon if they were able to answer the research questions and could shed light on our applied conceptualization of physicians’ SE. The findings are presented in three sections, corresponding to the three research questions. The findings in the first and third section are reported in narrative form to reflect our used methodology. The second section aimed to create an inventory of physicians’ self-regulations, and as such, we did not include quotations to reflect stories. We gave our participants fictitious names and refrained from other potentially traceable personal information to protect their identity yet facilitate the readability of the stories.

## Results

The first section elaborates on two characteristics of the employment context that interviewees perceived to affect their SE: group dynamics and a (mis)match between personal standards and prevailing group norms on professionalism. The second section describes interviewees’ self-regulations intended to sustain their employability. These self-regulations can be divided into three broader categories: (I) influencing work; (II) influencing themselves, and (III) influencing others. In the third section, two stories are presented that show consequences of self-regulation.

### The employment context affecting physicians’ SE

#### Group dynamics

Interviewees highlighted the importance of group dynamics for their ability to SE. Positive group dynamics were characterized by trust, support, a low threshold to ask for help and feedback, humour, social cohesion, group identity, and opportunities to ventilate, reflect and brainstorm with colleagues. Negative dynamics were described as regular or long-standing conflicts, prejudice in interactions between colleagues, ‘undercurrents’ that are felt but left unaddressed, belittling, bullying, favoritism, a focus on individual rather than group performance and perceived psychological unsafety. Group dynamics affected interviewees’ SE in several ways, both positively and negatively. First, positive group dynamics made work more *manageable.* For example, during the interview, Ezra explained how trusting one’s colleagues also extended to trusting them to take care of your patients when away from work, resulting in limited work-home interference. Ezra also explained that “having buddies” at work decreases the threshold for brainstorming about patients, which not only helps to share the heavy burden of medical responsibility but also generates better suggestions for patient care and counters one-sided “arrogant” decision-making. In another story, Sam’s spouse fell ill and eventually passed, but Sam stressed that the support of the group throughout, as well as their willingness to take over certain responsibilities, helped with persevering through that tragic period in life. Second, positive group dynamics made work more *enjoyable*. Ezra highlighted how laughing with colleagues helped to lighten one’s mood and Sam explained how incorporating some humor into work helped to put certain aspects of work into perspective:

*“Whenever I finished my consultations with patients, which are of course not always fun, and I am totally wrought up (…) because this patient drove me up the wall. Then I just, for a second, want to…Well, I walk back to our room—we have this big room with all the physicians together—and I talk to someone, usually the same one, and I tell my story, and we would laugh ourselves silly. (…) and after the story my colleague starts saying ‘Let me tell you what happened to me yesterday’. Isn’t that wonderful?”*

Third, positive group dynamics *facilitated learning*. Morgan and Xiao mentioned how positive group dynamics lowered the threshold to ask for help if you felt unskilled. Sam explained that when group dynamics are positive, people are more inclined to trust and accept feedback as genuine instead of a threat. Sam also experienced how “unconditional support” in a group created a kind of reciprocity between colleagues that enabled one to move beyond what they could achieve individually:

“You do not have to be friends, but you do have to trust each other, and you should not have different agendas, you must support each other. Unconditionally be there for each other. If you have that feeling, then you can move mountains.”

Fourth, negative group dynamics caused *apprehension to disclose* or resolve anything that may be relevant for one’s SE, whether it was personal problems, career ambitions, asking for feedback, or resolving conflicts. Morgan, who experienced favoritism by the boss to like-minded colleagues, explained how these dynamics caused apprehension to disclose issues, frustrations, and even ambitions due to a fear of coming across as a person who was not resourceful. Morgan regretted having never dared to mention the ambition and dream to advance a career in medical research. Izzy agreed that despite the potential of groups to realize ambitions, sharing those ambitions is not always perceived to be safe because certain people in groups may use that information to advance themselves.

*Izzy: “What are your priorities, what do you consider important? I think that everyone should at least disclose that to the group once a year (…). For example, if someone wants to develop themselves scientifically or want to become a professor, the group can help with that, but please disclose. It must be safe to do that though. If you do not have the feeling that it is safe, you will not.”*

*Interviewer: “and is it [safe]?”*

*Izzy: “No, it is not safe. Not safe enough. Because there are always a few that do have other agendas.”*

Fifth, negative group dynamics, where issues are not addressed or resolved, *created (long-term) distractions.* Both Noa and Morgan explained how experiencing negative interactions at work and refraining from discussing or resolving them cost considerable energy and headspace. Sixth, negative dynamics *inhibited learning* and professional development. Xiao, at some point, had an ambition to become an ‘excellent supervisor’ but was very disappointed when noticing a certain inertia from colleagues to help each other excel at work. Xiao mentioned how colleagues simply stayed behind their own desks and would brush off any questions or requests for help. 

Interviewees indicated that they associated positive dynamics with small or compact groups, fixed work locations, and (senior) colleagues or boards that are naturally emphatic or explicitly seek to improve or establish positive dynamics. Some physicians attributed negative group dynamics to larger groups (e.g., after a merger) or specific schedules (e.g., 24-hour shifts), where colleagues do not properly see, speak or acquaint with one another.

*“If you just concluded a merger [of different groups of physicians], people held more grudges and there is more envy. Which is a common thing if you mix different blood types, and I do not mean that you cannot mix blood types per se because it could work out fine, but nonetheless there was more suspicion, unawareness, especially when you physically work at different locations.” (Kelli)*

Interviewees also noted how negative dynamics may emerge because of increased turnover in departments, increasing rules and regulations, and changing priorities and role distributions among physicians—particularly young physicians—leading them to spend more time at home than in in the hospital. Several interviewees also realized how certain negative group dynamics were often the result of just a few people with the ability to set the tone in a group. Finally, many stressed in interviews how the COVID-19 pandemic facilitated more negative dynamics because groups no longer saw, spoke, or worked with each other physically.

***“****I said to the group that I worry about the dynamics, also because of COVID, obviously. Everything had to be done online and we did not see each other anymore, we were instructed to not be in the hospital if possible and if you were there to go straight home after you were done working. We started talking more about each other instead of with each other, and it truly was going the wrong way.” (Izzy)*

In sum, physicians perceive that positive and adverse group dynamics respectively affect physicians’ SE positively and negatively. Positively, as positive group dynamics can make patient workload or complexity more manageable, it makes work more enjoyable, and it facilitates learning among colleagues. Conversely, adverse group dynamics affect physicians’ SE, as they may prevent physicians from disclosing issues that can affect their SE (e.g., personal problems), create long-term distractions, and inhibit learning at work.

#### Normative mismatches

Interviewees also emphasized the significance of a (mis)match between personal standards and group norms on professionalism to their SE. Throughout interviews different forms of (mis)matches could be identified. First, interviewees perceived their personal professional standards to either align or differ from group or organizational norms for professionalism and quality of care. Second, interviewees perceived their personal professional standards to be higher than those of other colleagues or groups. Third, interviewees perceived difficulty living up to their own personal professional standards. Taylor, for example, reflected on the felt excitement and ambition to improve the quality of care for patients, perceived to be possible once working as a medical specialist with independence and autonomy over work-related decisions. Taylor explained, however, how this ambition clashed with the interests of established colleagues in the group, who were not keen on change, new initiatives, or improvements.

*“There were two older specialists, (…), I think that they were not at all excited about a young dog like me with a lot of qualities, who wanted to strive for higher quality standards, who was full of ideas. It was thwarted immediately: ‘No, we will do it like this’. (…) In that environment I was supposed to perform, and every quality improvement that I wanted to make, or truly anything I proposed, was immediately demolished*. (…) *It was not only a clash in terms of ‘I can’t spread my wings here’, but also ‘this is not my norm for quality performance.’”*

Xiao and Kit also reflected on their frustration with group norms for professionalism, which they perceived to be below (their own) standard. Both refer to a ‘six-minus’ [grade out of 10] culture, where scoring ‘average’ was deemed sufficient and any initiative to improve performance (e.g., reduce complications or readmissions) was nonexistent. Kit also observed that professional norms seem to differ between colleagues. Half of them seemed to be inquisitive and were willing to contribute (more) to the group, whereas the other half simply wanted to perform their day-to-day job and nothing else. Taylor reasoned that this partial inertia may be due to physicians not being able to deliver patient care in the way they would like, because of limited time, continuously full waiting rooms, administration, and focus on production rather than quality, making them feel exhausted, zoned out or disillusioned over time. Noa struggled with living up to her own professional standards and explained how a ‘good doctor’ ought to enjoy and be proficient at patient interaction and communication. Noa perceived not to possess this quality, problematized this and concluded that her functioning in practice is below standard. Relatedly, Sam explained to have observed how early-career physicians and residents often feel overwhelmed or feel that they have failed because of very high standards that they set for themselves and their colleagues, partially due to a culture of competition because of a scarcity of positions in certain specialties. Bo highlighted that high professional standards among younger physicians coincide with high standards for other activities in life (e.g., parenthood or sports), which adds to the difficulty of living up to all these standards.

“In these times, people put so much pressure on themselves. Back in the day, you were a resident and outside of that you have a bit of social life, and well, that was basically it. (…) Now? They have a 46-hour workweek including education, but they all decide to have children, wonderful of course, because that was the whole point, to be able to have children during residency and not after. However, in addition, they also obligate themselves to run the marathon in New York, to be a fantastic cook, and to make sure that their lives look great on Instagram. (…) And then they tell me: ‘But you have done that as well right?’. And I will be like, no, not at all. Not all at once like you.”

Interviewees also mentioned having experienced group/organizational norms that did match their own norms and standards. Morgan reflected on the energizing feeling in a previous job where the organization actively advocated for the ambition to combat cancer, which aligned with Morgan’s personal ambitions. The same applies for Kit and Nuri, who reflected on the pleasure of working with colleagues who functioned according to similar norms, values, and prioritization.

*“And so, Lane [colleague] and I, we were on the same page. We both had that old ‘Smith’ [previous head of department] mentality: patient care first and then all the fun stuff such as research, and yes, we maintained that. It was fun. Worked like a charm.” (Nuri)*

Whatever the form or reason, interviewees reported how mismatches—either from experience or by observing others—could result in job dissatisfaction, exhaustion, health problems, work-home interference, conflicts among colleagues, feelings of being an imposter or underperformer, sickness absence and turnover. In some instances, interviewees mentioned that they were socially isolated or excluded from groups when upholding or striving different professional norms. Taylor explained the consequences of a mismatch for her and her colleagues as follows:

“No, it is money. Truly, money. I am sure of it. They did not care about quality of care (…). Because of this, there were already so many colleagues who had complained but had dropped out. It is important to know that so many colleagues had already left because of this. So, there was this graveyard of people whom these men had left behind that simply could not continue to work with them (…).”

In sum, physicians perceive that different types of normative mismatches in their employment context affect their SE negatively because mismatches can over time lead to job dissatisfaction, health problems, imposter syndrome, conflicts at work, sickness absence and turnover.

### Physicians’ self-regulations to sustain employability

Physicians in this study tried to sustain their employability through self-regulation. These regulations can be divided into three categories. First, interviewees self-regulated by influencing their work. Second, interviewees self-regulated by influencing themselves. Third, interviewees self-regulated by influencing others. Self-regulations were not mutually exclusive or fixed. Interviewed physicians combined several self-regulations and changed these depending on the challenge or context at hand.

#### Influencing work

Interviewees self-regulated by influencing work directly when work practices were perceived to affect their SE. We identified four different self-regulations for this category. First, physicians tried to sustain their employability by *addressing* certain work practices. This could mean reporting to their superiors that certain aspects in the job affected their SE, confronting (difficult) situations and persons that affected their SE, being honest with patients or the board of directors when there were SE issues, and “fighting” to overturn certain work practices. Second, physicians tried to sustain their employability by *demarcating* work. They would circumvent or gradually discard unwanted or difficult aspects of the job, such as administration or difficult conversations with patients’ families. It could also mean not getting involved with other colleagues’ “dramas”, separating business and personal issues, not working overtime, working less contractually, setting and communicating personal boundaries, and clearly demarcating roles and responsibilities to others. This could also mean finding “your niche” or “the fit” in subspecialties or tasks to sustain one’s employability. Third, interviewees tried to sustain their employability by *extending* their work. This could mean that interviewees engage in extra roles, such as management, committees, quality improvement initiatives, research, and training residents, usually outside of their normal working hours or responsibilities. Some mentioned extending work to live up to standards of how a physician should function adequately, to find challenge or meaning in work, learn other skills beyond clinical work and feel energized. Sometimes it was also a strategic self-regulation to broaden their sphere of influence and as a backup plan in case clinical work would no longer be possible in the future. Fourth, and perhaps its most extreme form, interviewees regulated work by *changing jobs* and started working for other organizations or became self-employed.

#### Influencing themselves

Instead of trying to influence work practices directly, physicians in this study also regulated themselves to sustain their employability. Seven self-regulations could be identified for this category. First, interviewees tried to sustain their employability by *accepting* certain work practices. This would mean that physicians were able to put certain characteristics or situations at work into perspective, to not take everything personally, to accept what was in and out of their sphere of influence, to be able to let things slide, and to decide to no longer care about “trivial” things. Acceptance could also mean conforming to certain work practices to avoid conflict or reputation loss. Second, interviewees influenced themselves by *changing* themselves. This ranged from the very subtle, such as reflecting on one’s share and pitfalls in certain problematic situations and adapting behavior accordingly, to the extreme, where one physician acknowledged “putting on a masque” or playing a role at work to be able to sustain one’s employability over time. Third, interviewees tried to sustain employability by *creating independence*. This could entail financial independence so one could always quit their job without any financial consequences, or more of a mental independence, where interviewees tried to not let their happiness or identity depend too much on their work. Fourth, interviewees sustained their employability by *harnessing motivation*, often a deep-rooted intrinsic one, to persevere and cope with adverse situations at work. Here interviewees would remind themselves of why they had become physicians in the first place or consciously recall moments that had brought joy in work. Fifth, interviewees influenced themselves by *seeking support* and feedback from others, usually from a coach, other (healthcare) professionals, mentors, and colleagues. Physicians would seek support to cope with work-related affairs and personal circumstances that affected, or threatened to affect, their SE. Sixth, interviewees tried to sustain their employability by *engaging in self-care.* They do so by taking care of themselves through exercise and proper diet, by taking the time to detach from work, and to relax and to (re)connect with themselves through hobbies or mindfulness. Seventh, interviewees sustained employability by *educating themselves*. This could mean soaking up colleagues’ best practices, learning about health care from different disciplines, following an MBA in health care, reading up on the latest best evidence, and following courses on work-related topics, such as group dynamics.

#### Influencing others

Some physicians tried to sustain their employability by influencing others. We identified four different methods here. First, physicians mentioned how they were able to cope with certain work practices by *assembling* the right people around them. This could either mean influencing recruitment processes, building an alliance with like-minded people, finding one’s own replacement before retirement, or teaming up with colleagues to address issues at work. Sometimes assembling also meant consciously sidelining or circumventing people. Second, physicians tried to sustain their employability by *connecting* with other physicians. This could mean that they acknowledged that they needed others to function adequately, which in turn required involving other departments or patient associations. It also meant building and investing in relationships with people at work that would make work more enjoyable and bearable, would facilitate feedback, and that could open doors to realize ambitions and dreams. Third, physicians stressed the importance of *empowering* others to sustain their own employability, as this could lead to a return on investment. In practice, this often meant providing opportunities to others, helping them, sharing acquired wisdom from experience, or educating residents/interns. Fourth, some physicians in this study self-regulated by *strategically positioning* themselves vis-à-vis others. This included being explicit on (own) roles, expectations, and sentiments vis-à-vis others. Others mentioned strategically involving “narcissistic” colleagues in certain ways to create goodwill, which in turn made working with them easier in the future. 

To conclude, this section has demonstrated that physicians used many ways to self-regulate their SE throughout their careers. These self-regulations can be categorized into three types of self-regulations: those influencing work, those influencing themselves or those influencing others to sustain their employability. Self-regulations were neither stable nor mutually exclusive. Physicians reported adjusting self-regulations over the course of their careers depending on the situation or context at hand and seemed to use different types of self-regulations at the same time.

### Consequences of self-regulations

Interviewees sometimes reflected on the consequences of their self-regulations. They contemplated whether self-regulations had yielded the intended results for their SE, and to what extent self-regulations were sustainable to use in the long run. Moreover, interviewees reflected on unforeseen or unintended consequences of their self-regulations for the employment context in which they and their colleagues had to continue performing. In the following section, we present two stories in which physicians had realized the impact of their self-regulations, and where they made a conscious effort to either intensify or adjust their self-regulations to consider these implications. These stories are not meant to serve as a blueprint for which self-regulations work in practice. Instead, these stories were chosen because they show the interrelations between the employment context, physicians’ SE and their self-regulations.

#### Nuri’s story

Nuri starts with a reflection on the residency period, where he acknowledged to have been blessed by very positive group dynamics at work. Nuri explains how solidarity, fun, and having senior role models at work made hard work and long hours more manageable.

*“Yes, it was an incredibly busy time, you would have shifts every other day. The weekend shift started on Friday mornings and ended Monday evenings. It was extremely hard work, but I also had a lot of fun. (…). We had a very nice group. There was a big sense of solidarity.”*

Moreover, Nuri explained how he had been actively mentored by colleagues during this period, which enabled him to uncover and develop talents and qualities that benefitted his ability to function adequately at work during the rest of his career. Nuri, who eventually became head of a department, explained how he had come to realize the reciprocal effect of investing in others, both for himself and for the group. As a result, he actively and consciously set out to create an amicable work environment by treating colleagues as equals, showing kindness and respect, and *connecting* with others. Nuri explained how he acknowledged the importance of *empowering* colleagues to cultivate their individual talents and to *assemble* diverse teams that would enable “cross-fertilization” and learning between colleagues. He also mentioned never having refrained from helping with patient care, regardless of his position or who was asking.

“It is important to realize that you must help each other. It is something I have looked at very consciously. You must empower one another. For example, Joni, who was brilliant, technically. Still gives me goosebumps. So, you will have to let him operate on these types of patients. When it became clear that he wanted to stay until retirement, we did encourage him to obtain his doctorate, because otherwise people would walk all over him, because let?s be honest, that?s how it goes in the academic world. And so, we gave him time, and he wrote a beautiful dissertation (…). And Theo, much more the researcher and a bit of a loner. It was kind of hard having to work with him, but he was just good. Also, he was extremely honest and couldn?t stand injustice. So, when I would treat people wrongly, he would always come to my room and tell me to stop it and to go and talk to that person and admit having acted wrongly. (…). We would all treat each other respectfully and I have appreciated that enormously. (…). And that is why you should empower everyone.”

Nuri believed that investing in relations at work had provided him with much goodwill, genuine feedback on his performance, and smooth collaborations that, in turn, aided him in functioning adequately in his position as head of department.

“Robin [former role model in residency] did have complications sometimes. He would just be honest about it, nothing mysterious. (…) and when it got too complicated, he would call in someone to help. I have always kept that in mind. And later, when you become stiffer, which can happen when you?re older (…), I would simply call Joni or Theo to help a hand. After all, I had always learnt that that was normal. (…) You must be able to perform in an environment of trust and once you are the boss, you need goodwill. But you can promote trust by just working alongside them. Doing the same shifts, the chores. (…) A boss in this profession that does not actively take part will be in for a difficult job, whether that be today or tomorrow, it will become hard.”

Nuri seemed to have mostly influenced others, and in turn, his work environment, to sustain his own employability, which, in his perception, improved his ability to function adequately as head of a department. Moreover, these self-regulations, especially as these were enacted by the head of department, may also have acted as signals and examples to other physicians in the group to adopt similar self-regulations.

#### Xiao’s story

Xiao started by describing how demanding and time-consuming physicians’ jobs are where many nonclinical tasks are not being compensated for time or salary, but physicians are nonetheless expected to excel at.

”All of this means that you are still performing according to an older culture, where medical specialists were supposed to and expected to work, day and night, and in weekends. And everything you cannot finish during working hours you have to do in your own time.”

Xiao went on to explain how at first these expectations and job demands were met by *extending* work, more specifically by investing a great deal of personal time into work, also because he felt it was important and rewarding to engage in these extra roles. However, Xiao acknowledged how unsustainable this self-regulation was in the long run because it deteriorated his personal health and time with his family. Xiao also explained how he had come to realize that extending one’s work to live up to his own professional standard, also affected colleagues, because, by doing so, he contributed to the normalization of working overtime. He realized that he not only was normalizing excessive job demands but was also normalizing how to cope with excessive job demands (i.e., *extending* work), despite the negative consequences he himself had experienced.

“We do a lot of things. I think (…) from the conviction that we think it is important, fun, because we think we must, because it is important for the patient, because it makes our work more fun, interesting, and because it makes health care safer. But at the same time, we experience that this requires an unbelievable amount of time. [after consulting other colleagues] I thought ‘Oh, I am not alone, apparently, we all deal with this, but that is not ok. However, in the end, it is also us who accepts it.”

After realizing the negative consequences of his self-regulations, Xiao started *demarcating* work more. He decided to invest only normal and paid working hours, rather than personal time, into quality improvement initiatives. Even if this meant that these projects would take longer to finish than before.

“So that is what I said to the head of my department. I love to work hard, I consider my projects very important, I see that the projects matter and are useful, and that they entail everything to improve health care, both for patients and professionals. But also, how these projects take an unbelievable amount of time and that I was no longer planning to invest my own family time into these projects, and rather use my own time to simply recover from work. Of course, there are exceptions, occasionally, you simply must work overtime. But it should remain exceptional. So now, most days, and weekends that I am off, I am completely off. And my supervisor looked at me and said: ‘you are totally right, I want to implement that as well’, and he admitted to doing the exact same thing, working way too much in the evenings and weekends, but that he would want to create a culture where that would not be considered normal.”

The last quote illustrates how Xiao, after becoming aware of the long-term and wider consequences of his self-regulations, adjusted his self-regulations in a way that considered both his own and others’ SE.

Both Nuri’s and Xiao’s stories illustrate how physicians’ self-regulations affected their SE (e.g., how Nuri’s willingness to help others created goodwill enabling him to function more adequately) and the employment context (e.g., when Xiao recognized that extending work tasks and hours may have normalized this to others in his employment context).

## Discussion

This study examined physicians’ perceptions of their employment context and how it affects their sustainable employability (SE), their self-regulations intended to sustain employability, and the consequences of these self-regulations.

### Key findings

First, our results identify two group-level factors within the employment context that physicians perceive to affect their SE: group dynamics and normative (mis)matches within or between physicians. Positive group dynamics enhance physicians’ SE by promoting manageability, enjoyment and learning. Adverse group dynamics impede physicians’ SE by discouraging issue disclosure, creating distractions and hindering learning. Research on disruptive physician behaviors corroborates these findings. Disruptive behaviors, such as bullying, harassment, and favoritism—which interviewed physicians described as ‘adverse group dynamics’ - have also been found to discourage staff from seeking help or sharing best practices due to fear of retaliation [8]. Over time, these behaviors erode teamwork and camaraderie [8], which are arguably crucial ingredients for physicians to function adequately at work (i.e., their SE). Another contextual factor - normative mismatches—affects physicians’ SE, as these create job dissatisfaction, health problems, conflicts, imposter feelings, social isolation, sickness absence and turnover. Our findings can be interpreted by social identity theory [63]. Motives to dissent and deviate from group norms include disengagement, loyalty (i.e., to protect groups), strong personal moral convictions, aspiring individuality, and tangible gains [64]. Our findings suggest that physicians’ personal professional standards, often derived from moral convictions, are the basis for perceived normative mismatches. Moreover, normative mismatches may indeed be met by group responses, such as isolation and exclusion of ‘deviants’, or alternatively ‘deviants’ leaving their groups [65]. This may be unfortunate, as ‘norm violators’ may also positively affect group functioning and performance [66]. Nonetheless, prolonged normative mismatches may result in moral distress [67], and, when left unexpressed or unresolved, may leave physicians disillusioned [68]. Both adverse group dynamics and normative mismatches may negatively affect physcian’s SE through perceived person-environment misfit and value incongruence [11, 36]. Second, this study presents an inventory of self-regulations that physicians enact when trying to sustain their employability. These self-regulations can be categorized into three categories: self-regulations pertaining to influencing work, oneself, or others. The categories identified in this study correspond to ways in which individuals craft their jobs, which can be task (i.e. work), cognitive (i.e. oneself), or relational (i.e. others) [69]. Third, this study revealed that self-regulation may have different consequences for physicians’ SE in the short and long term and that self-regulation may also unintentionally influence or shape pysicians’ employment context, sometimes in a dysfunctional manner. Bakker and de Vries provide a useful model that distinguishes between maladaptive and adaptive self-regulation and how both emerge and continue to be used [39]. When employees experience increased job strain, they are more likely to enact maladaptive self-regulation (e.g., undermining oneself), which continues to increase their job demands, job strain and the likelihood of combatting these issues with maladaptive self-regulation (i.e., loop A). Employees who are not strongly strained by work have more conserved energy to engage in adaptive self-regulation, such as job crafting (categorically requiring energy). Adaptive self-regulations decrease job demands and increase job resources, resulting in less job strain and again increase the likelihood that employees continue to engage in adaptive self-regulation (i.e., loop B) [39]. It may be that persistent straining contextual factors in physicians’ employment context may make it increasingly difficult for physicians to continue enacting adaptive self-regulation, forcing them to gradually resort to maladaptive self-regulations (i.e., misregulation) that further impede their SE.

### Synthesis of findings

**Fig. 2:**
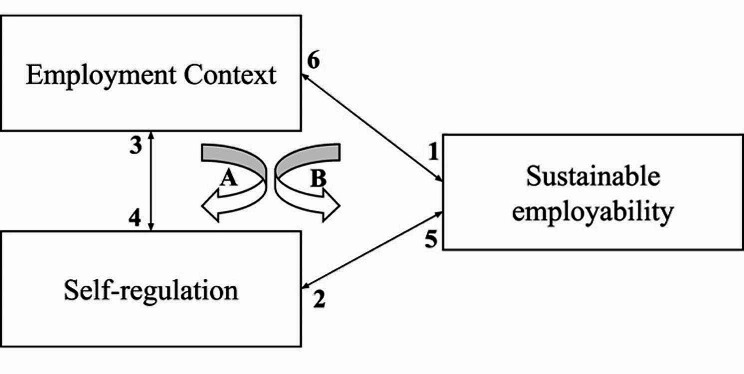
A cyclical conceptual model of physicians’ SE

From our key findings, this study distills that a physician’s SE develops from the interplay between the employment context in which physicians function *and* physicians’ self-regulations to sustain their employability. Figure 2 visualizes how physicians’ SE is dynamic and cyclical rather than a fixed trait that remains constant throughout careers. From the empirical findings and known literature we present two loops with corresponding propositions that interrelate physicians’ employment context, their self-regulation, and their SE.

Loop A generates propositions 1, 2, and 3. One, physicians’ SE is affected by different-level (i.e. personal, group, organizational, societal) factors in the employment context [1]. Two, diminished SE incites a motivational mechanism in which physicians enact self-regulations to alleviate deviations from the standard (here: adequate functioning at work/SE). Three, self-regulations, whether intended or not, may change or reinforce existing work practices in the employment context, which in turn may continue to affect employees’ SE. Loop B generates propositions 4, 5, and 6. Four, self-regulations are often context-specific processes that physicians develop and initiate because they align with prevailing norms, expectations, and social identities [50]. Five, self-regulations can affect physicians’ SE positively or negatively, and this effect may be different in the short or long term. Six, changes in SE may bring about changes in employment characteristics (e.g., job loss) [1].

### Theoretical implications and future research

Considering physicians’ SE to develop from the employment context and their self-regulations avoids isolated and simple conclusions that either criticize specific work practices in the employment context or stigmatize physicians’ self-regulations. After all, physicians’ self-regulations may be “normal and understandable reactions to an otherwise unmanageable situation” [12], and certain work practices, although perceived as dysfunctional by physicians for their SE, may remain “necessary evils” for the greater good in health care [70]. Instead, researchers may continue to explore how self-regulations or specific employment characteristics develop and institutionalize over time. We propose that understanding physicans’ SE as contextual and cyclical is more interesting and constructive. Future research may continue to explore this train of thought by formulating and testing specific propositions derived from our cyclical conceptual model. Moreover, from the interviews, it seems that the development of physicians’ self-regulations dates back to experiences in medical internships and residency. Research has described how, during these important socialization years, the ‘hidden curriculum of medicine’, or the uncodified rules “that concern how clinical thinking and performance (…) [and] the way physicians are supposed to act professionally and collegially”, are passed on [51]. Future research may explore whether self-regulations are indeed shaped during formal education and to what extent self-regulations form as a result of traditional and/or modern convictions of physicians [70].

### Implications for practice

Self-regulation is argued to be a skill or competence that can be trained and nurtured by both physicians and their environment [71]. Consequently, physicians may use the inventory of self-regulations to reflect whether they recognize regulations as their own, to what extent these self-regulations are functional to their SE in the immediate and long run, whether self-regulations may feed back into the employment context, to what extent this is considered desirable, and if not, whether it is within their sphere of influence to adapt these self-regulations. Moreover, as individual physician’s SE is so context dependent, groups of physicians, their employers, and professional bodies, could use our findings to examine and discuss to what extent normative mismatches and adverse group dynamics affect physicians’ SE in their contexts. Furthermore, medical educators and supervisors may use the important socialization years during medical internships and residency to instill more healthy and sustainable self-regulations for young physicians [72]. Finally, regulating agencies in health care may use our findings to understand how physicians’ SE issues may gradually originate from a dysfunctional context and/or maladaptive self-regulation. Context and self-regulating behavior may be potential (additional) targets for intervention by regulators to contribute to physicians’ SE.

### Strengths and weaknesses

This research provides an in-depth representation that respects the complexity of the reality in which physicians function. We argue that this study has a few important strengths. First, narrative inquiry enabled the construction of a detailed, subjective, and uncodified reality that would have been hard to grasp when conducting more traditional qualitative or quantitative research. The themes resulting from this inquiry can be considered valid and potentially universal because they were evident *despite* detailed differences in experiences and contexts. Nonetheless, this research was predominantly exploratory in nature, and due to the nature of our sampling strategy, we cannot generalize our findings to all physicians. Future research may replicate our approach for stratified physician samples to determine whether important demographic, specialty, and/or tenure differences exist. Second, we believe that the interaction between the interviewees and the interviewer, due to her similar background, elevated narrative interviewing because it enabled both sides to share relevant personal stories. This, in turn, seemed to establish more rapport, frankness and depth to participants’ stories. Third, narrative inquiry also seemed to function as an opportunity for physicians to reflect and learn from experiences and self-regulations, which contrasts with practice where there is hardly any time or support to ‘zoom-out’ according to some of the participants. One limitation of this study is that it is limited to the Dutch context. Although the organization of physician work is not drastically different between countries, the way in which health care is organized, financed, and regulated in the Netherlands also constitutes part of the employment context that likely affects physicians’ SE. Future research may replicate our study in different contexts to determine whether our findings hold.

## Conclusion

During their careers, physicians navigate complex group dynamics and normative mismatches regarding professionalism that affect their SE. To sustain their employability, physicians self-regulate by influencing their work, influencing themselves and influencing others. Physicians’ self-regulation may have unintended and/or dysfunctional consequences for their own SE and their employment contexts. Insights from this study can be used to understand and appraise how physicians try to navigate and mitigate complex challenges at work and to prevent dysfunctional work practices *and* dysfunctional self-regulations.

## Data Availability

Interview transcripts are not publicly available to protect the privacy and confidentiality of study participants. However, the data supporting the analysis (codes and underlying (anonymized) quotes) may be available from the corresponding author (IV) upon reasonable request.

## Declarations

### Authors’ information

IV is a social scientist with a master’s degree in Learning & Development and Occupational Health and Safety. IG is a general surgeon also employed as an assistant professor on safety in healthcare. IL is a nonpracticing physician with an extensive background in patient safety and regulation. JW is a health services researcher with a background in professional performance and quality and safety of healthcare.
